# Major cardiovascular and bleeding events with long-term use of aspirin in patients with prior cardiovascular diseases: 1-year follow-up results from the Management of Aspirin-induced Gastrointestinal Complications (MAGIC) study

**DOI:** 10.1007/s00380-019-01484-0

**Published:** 2019-08-24

**Authors:** Shinichiro Uchiyama, Shinya Goto, Hideki Origasa, Naomi Uemura, Kentaro Sugano, Hideyuki Hiraishi, Kazuyuki Shimada, Yasushi Okada, Yasuo Ikeda, The MAGIC Study Group, Yasuo Ikeda, Shinichiro Uchiyama, Yasushi Okada, Kazuyuki Shimada, Shinya Goto, Kentaro Sugano, Hideyuki Hiraishi, Naomi Uemura, Hideki Origasa, Naomi Uemura, Takashi Kawai, Shinichi Nakamura, Chouitsu Sakamoto, Hidekazu Suzuki, Shinichiro Uchiyama, Yasushi Okada, Kazuyuki Shimada, Shinya Goto, Saichi Hosoda, Yukito Shinohara, Toshifumi Hibi, Hiroko Usami

**Affiliations:** 1 Clinical Research Center for Medicine, International University of Health and Welfare, Center for Brain and Cerebral Vessels, Sanno Hospital and Sanno Medical Center, 8-5-35 Akasaka, Minato-ku, Tokyo, 107-8332 Japan; 2 Department of Medicine (Cardiology), Tokai University School of Medicine, Isehara, Kanagawa Japan; 3 Department of Biostatistics and Clinical Epidemiology, University of Toyama School of Medicine, Toyama, Japan; 4 Department of Gastroenterology and Hepatology, Kohnodai Hospital, National Center for Global Health and Medicine, Chiba, Japan; 5 Department of Medicine, Division of Gastroenterology, Jichi Medical University, Shimotsuke, Tochigi Japan; 6 Department of Gastroenterology, Dokkyo Medical University, Mibu, Tochigi Japan; 7 AOI Nanasawa Rehabilitation Hospital, Atsugi, Kanagawa Japan; 8 Department of Medicine, Division of Cardiology, Jichi Medical University, Shimotsuke, Tochigi Japan; 9 Department of Cerebrovascular Medicine and Neurology, National Hospital Organization Kyushu Medical Center Clinical Research Institute, Fukuoka, Japan; 10 Graduate School of Advanced Science and Engineering, Waseda University, Tokyo, Japan

**Keywords:** Aspirin, Cardiovascular disease, Cardiovascular events, Hemorrhagic events, Registry

## Abstract

Aspirin should be used for the prevention of cardiovascular (CV) events by the risk–benefit balance. This study was conducted to clarify CV and bleeding events in Japanese aspirin users with a history of CV diseases. This study was a prospective, nationwide, multicenter cooperative registry of Japanese patients with CV diseases at risk of thromboembolism who were taking aspirin (75–325 mg) for at least 1 year. We observed major CV and bleeding events during follow-up. Patients with history of ischemic stroke (IS), transient ischemic attack (TIA), coronary artery disease (CAD), atrial fibrillation (AF), and venous thromboembolism (VTE) were included and analyzed in this sutdy. CV events included IS, TIA, CAD, CV death, angioplasty or stenting, and hospitalization because of CV disease. Bleeding events included major bleeding requiring hospitalization and/or blood transfusion. A total of 1506 patients were categorized into IS/TIA (*N* = 540), CAD (*N* = 632), and AF/VTE (*N* = 232). Among them, 101 patients had two or more categories. CV and bleeding events occurred in 61 (3.82%/year) and 15 patients (0.93%/year), respectively. The annual rates of CV and bleeding events were 2.81% and 0.93% in IS/TIA, 5.32% and 0.75% in CAD, 1.15% and 1.15% in AF/VTE, and 6.44% and 0.91% in two or more disease categories, respectively. The Management of Aspirin-induced Gastrointestinal Complications (MAGIC) study clarified the rates of major CV and bleeding events with long-term use of aspirin in patients with prior CV diseases in real-world clinical practice. The risk–benefit balance of aspirin was acceptable in patients with IS/TIA, CAD, and multiple CV diseases but not in those with AF/VTE.

Trial Registration: The MAGIC Study is registered at UMIN Clinical Trial Registry (www.umin.ac.jp/ctr/index-j.htm), number UMIN000000750.

## Introduction

Aspirin has been established to reduce the risk of cardiovascular (CV) events, including stroke, myocardial infarction (MI), and vascular death, in patients with a history of CV diseases [1, 2]. However, aspirin increases the risk of gastrointestinal (GI) injury, which may lead to GI bleeding, even at a low dose [3]. Therefore, the use of aspirin should be determined using the risk–benefit balance [4]. Aspirin can be recommended in cases having a significant net clinical benefit estimated by reducing ischemic events and increasing hemorrhagic events.

The American Heart Association recommends the use of low-dose aspirin (75–325 mg) for patients having a 10-year CV event risk of ≥ 10% [5]. The US Preventive Services Task Force recommends prophylactic aspirin therapy only in patients with a 5-year CV risk ≥ 3% [6]. However, the risk of CV events is decreasing owing to the recent progress in risk factor management and may differ between ethnicities [7].

CV and hemorrhagic events were investigated during a 1-year follow-up period in Japanese patients receiving low-dose aspirin for the prevention of CV events enrolled in the Management of Aspirin-induced Gastrointestinal Complications (MAGIC) study.

## Methods

### Study design

The MAGIC study was a nationwide, multicenter-cooperative, prospective, observational study conducted in Japan. Details of the study design are described elsewhere [8]. Participants were enrolled from multiple disease categories, including those with coronary artery disease (CAD), ischemic stroke (IS), transient ischemic attack (TIA), atrial fibrillation (AF), and venous thromboembolism (VTE) requiring antithrombotic therapy, and recruited from 63 sites across the regions of Japan between April 2007 and September 2009. CV and hemorrhagic events as outcomes predefined in the protocol were evaluated by investigators at individual sites [8]. The study protocol was approved by the institutional review board at each site. All participants signed written informed consent forms. The study design was formulated by the Organization Committee (see “Acknowledgements”: A complete list of the MAGIC study group), and data were collected through a Web-based system [8].

### Study population

The inclusion and exclusion criteria were described elsewhere in detail [8]. Details of the background characteristics and risk factor profiles of the patients recruited at baseline are described elsewhere [9]. The incidence rates of major vascular and bleeding events in patients with CV diseases who were taking aspirin (75–325 mg daily) and followed up for 1 year were analyzed in this study.

Underlying CV diseases were categorized into IS/TIA, CAD, and AF/VTE. CAD included myocardial infarction (MI) and angina pectoris.

The baseline data included sex, age, body height, body weight, body mass index, vascular risk factors including hypertension, dyslipidemia, diabetes mellitus, current cigarette smoking and alcohol drinking, use of aspirin (dosage and formulations: buffered or enteric coated), and concomitant use of other drugs.

### Outcomes

CV events were judged by investigators in each site [8]. The incidence rates of CV events, including IS, TIA, CAD, CV death, coronary or other angioplasty or stenting, and hospitalization because of CV disease and bleeding events, or blood transfusion during the 1-year follow-up period were analyzed in this study.

### Statistical analysis

The results were expressed as mean ± standard deviation. The incidence rates and 95% confidence intervals (CIs) of bleeding and cardiovascular events were calculated using the Poisson method. All analyses were performed using R 2.15.1 (R Foundation for Statistical Computing, Department of Statistics and Mathematics, Wirtschafts University, Vienna, Austria). For all hypotheses, *p* values < 0.05 (two-tailed) were considered significant.

## Results

**Fig. 1:**
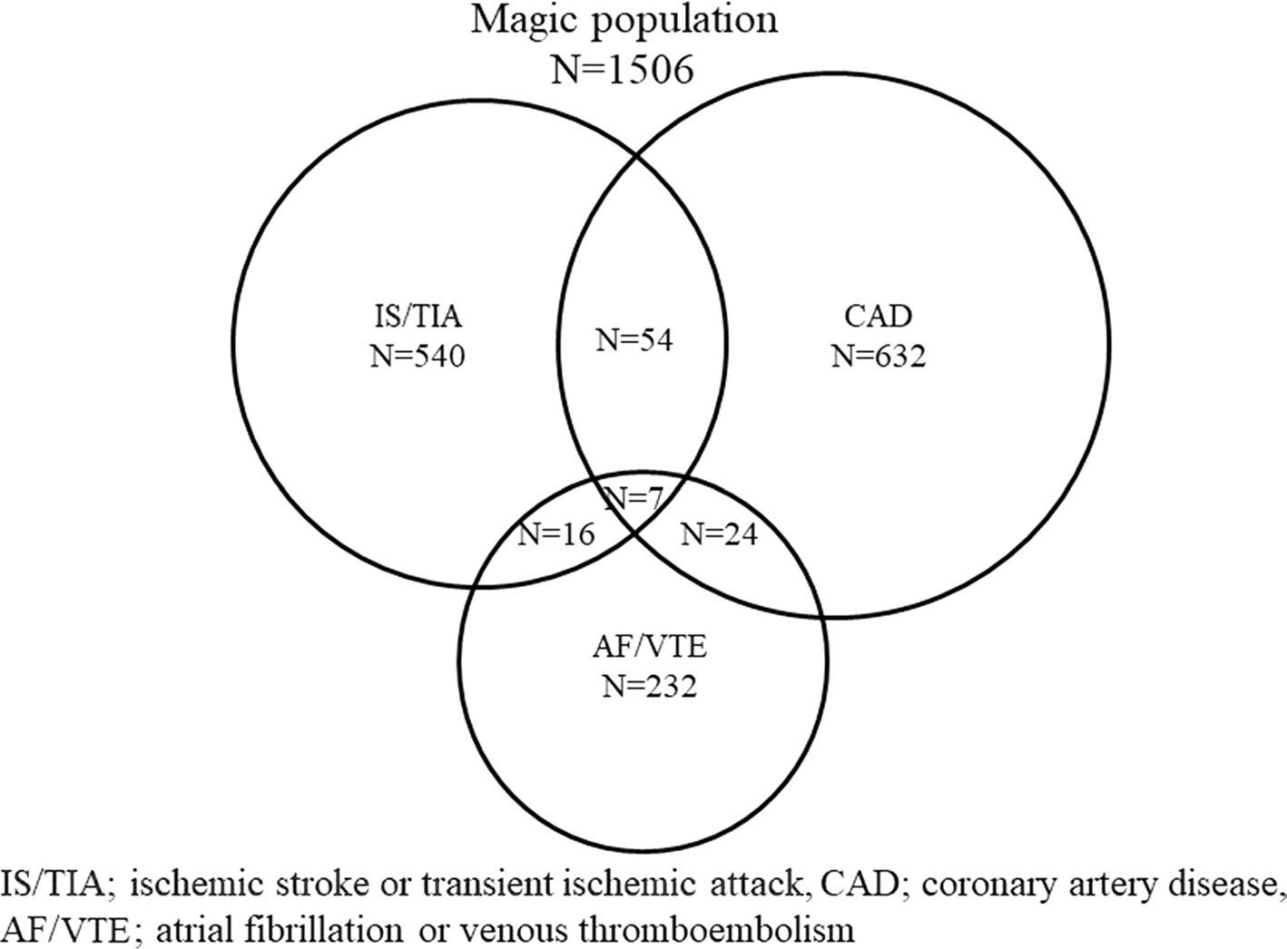
MAGIC population profile. *IS/TIA* ischemic stroke or transient ischemic attack, *CAD* coronary artery disease, *AF/VTE* atrial fibrillation or venous thromboembolism

The baseline data on background characteristics, risk factors, and use of aspirin and other concomitant drugs are shown in Table 1. A total of 1506 patients were classified into the three disease categories, namely, IS/TIA (*N* = 540), CAD (*N* = 632), and AF/VTE (*N* = 232), on the basis of the underlying disease at baseline (Fig. 1). Among the patients, 61 had IS/TIA and CAD, 23 had IS/TIA and AF/VTE, 31 had CAD and AF/VTE, and 7 had all of IS/TIA, CAD and AF/VTE.

**Table 1:** Characteristics of the patients at baseline

Characteristic	Mean ± SD or number (%)
Men (%)	1108 (73.6%)
Age (year)	68.2 ± 9.5
Body height (cm)	161.4 ± 8.4
Body weight (kg)	62.6 ± 11.1
BMI	23.9 ± 3.2
Hypertension (%)	1077 (71.5%)
Dyslipidemia (%)	848 (56.3%)
Diabetes mellitus (%)	424 (28.2%)
Current cigarette smoking (%)	158 (10.5%)
Alcohol drinking (%)	606 (40.2%)
Dose of aspirin (mg)	103.9 ± 26.0
Use of NSAIDs (%)	80 (5.3%)
Use of other antiplatelet drugs (%)	368 (24.4%)
Use of Warfarin (%)	179 (11.9%)
Use of antihypertensive drugs (%)	1124 (74.6%)
Use of lipid lowering drugs (%)	779 (51.7%)
Use of antidiabetic drugs (%)	291 (19.3%)
Use of antiarrhythmic drugs (%)	212 (14.1%)
Use of antiulcer drugs (%)	785 (52.1%)

*SD* standard deviation, *BMI* body mass index, *NSAIDs* non-steroidal anti-inflammatory drugs

**Fig. 2:**
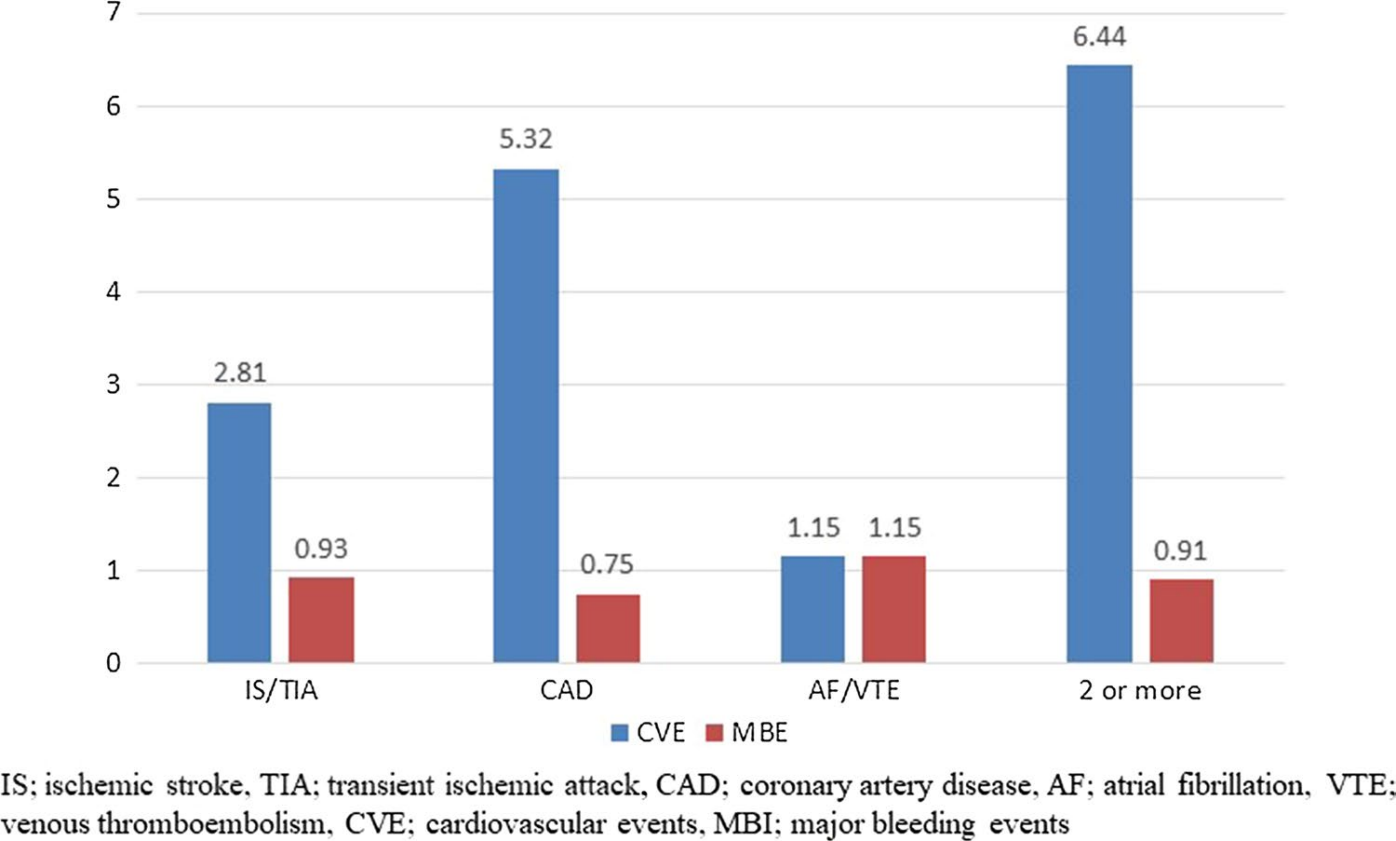
Vascular and hemorrhagic events in 4 categories of patients. *IS/TIA* ischemic stroke or transient ischemic attack, *CAD* coronary artery disease, *AF* atrial fibrillation, *VTE* venous thromboembolism, *CVE* cardiovascular events, *MBI* major bleeding events

A total of 1506 patients were followed up for a mean of 390 days. CV events occurred in 61 patients (3.82%/year; 95% CI 2.97–4.91%/year), and bleeding events occurred 15 patients (0.93%/year; 95% CI 0.57–1.54%/year) (Table 2). According to the disease categories at baseline, the annual incidence rates of CV and bleeding events were 2.81% and 0.93% in IS/TIA, 5.32% and 0.75% in CAD, 1.15% and 1.15% in AF/VTE, and 6.44% and 0.91% in two or more categories of diseases, respectively (Fig. 2).

**Table 2:** Major vascular and bleeding events

Major vascular and bleeding event	Number (%/year, 95% CI)
Cerebrovascular or Cardiovascular event	61 (3.82%, 2.97–4.91%)
Nonfatal stroke or transient ischemic attack	11 (0.68%, 0.38–1.22%)
Nonfatal myocardial infarction	5 (0.31%, 0.13–0.73%)
Coronary angioplasty or stenting	29 (1.81%, 1.26–2.60%)
Hospitalization due to other vascular event	14 (0.87%, 0.52–1.46)
Cardiovascular death	2 (0.12%, 0.03–0.45%)
Major bleeding	15 (0.93%, 0.57–1.54%)
Cerebral bleeding	4 (0.25%, 0.01–0.64%)
Gastrointestinal bleeding	10 (0.62%, 0.34–1.14%)
Upper gastrointestinal bleeding	1 (0.06%, 0.01–0.35%)
Lower gastrointestinal bleeding	9 (0.56%, 0.29–1.06%)
Other bleeding	1 (0.06%, 0.01–0.35%)

## Discussion

In our MAGIC study, the incidence rate of CV events was 3.82%/year and the incidence rate of bleeding events was 0.93%/year (cerebral hemorrhage, 0.25%/year and GI bleeding, 0.62%/year). According to a meta-analysis by the Antithrombotic Trialists’ Collaboration (ATT), the annual incidence rate of vascular events, including MI, stroke, and vascular death, was 8.19% in the controls and 6.69% in patients who were receiving aspirin for secondary prevention of vascular disease [2]. The incidence rate of CV events in the MAGIC study was than that in the aspirin group of ATT. On the other hand, the incidence rates of bleeding events in the MAGIC study were comparable to those reported in the previous studies conducted in patients with a history of CV disease [2, 10, 11].

In the Clopidogrel versus Aspirin in Patients at Risk of Ischaemic Events (CAPRIE) trial, the annual risk of stroke, MI, and vascular death was 5.83%, whereas the annual risks of intracranial hemorrhage and GI bleeding were 0.47% and 0.72%, respectively (any severe bleeding disorder was 1.55%), in 9586 patients with ischemic stroke, MI, or peripheral vascular disease who were treated with aspirin (325 mg/day) [10]. In the Clopidogrel for High Atherothrombotic Risk and Ischemic Stabilization, Management and Avoidance (CHARISMA) trial, the rate of the primary efficacy end point, which was a composite of MI, stroke, and death from CV causes, was 7.3%, and the incidence rate of severe bleeding was 1.3%. The incidence rate of primary intracranial hemorrhage was 0.3%,, but the incidence rate of GI bleeding was not documented with aspirin (75–162 mg/day) alone in 7801 patients with clinically evident CV disease or multiple risk factors [11]. In a subanalysis of CHRISMA on bleeding complications, severe bleeding was observed in 102 patients (1.3%) who were receiving aspirin alone [12].

The lower risk of CV events in the MAGIC study may suggest the recent progress of risk factor management. However, the risk of CV events was still high enough for aspirin use in our patients with IS/TIA and CAD, on the basis of the risk–benefit balance, as shown in Fig. 2 [5-7]. On the other hand, the incidence rates of CV and bleeding events were the same in our patients with AF/VTE, as shown in Fig. 2. Namely, CV risk was lower in the patients with AF/VTE than in those with IS/TIA, CAD, and multiple diseases, while the bleeding risk in the patients with AF/VTE was comparable with those in the other patient categories. Therefore, our results suggested that aspirin is not recommended for the prevention of CV events in patients with AF/VTE.

Most AF patients recruited to the MAGIC study were unsuitable or unwilling to receive warfarin or at a low risk of stroke. According to the results of the AVERROES (Apixaban Versus Acetylsalicylic Acid [ASA] to Prevent Stroke in Atrial Fibrillation Patients Who Have Failed or Are Unsuitable for Vitamin K Antagonist Treatment), stroke or systemic embolism occurred more frequently in the aspirin group than in the apixaban group, and major bleeding occurred equally in both treatment groups among patients unsuitable to receive vitamin K antagonists and at risk for stroke [13, 14]. Therefore, aspirin showed no potential for stroke prevention in AF patients. In reality, guidelines of the European Society of Cardiology do not recommend aspirin use under any condition for stroke prevention in AF patients [15]. With aspirin, CV events are difficult to prevent in patients with VTE. According to the Cochrane Database Systematic Review, aspirin did not reduce recurrent VTE, VTE-related mortality, stroke, or MI as compared with placebo for extended prophylaxis in patients with unprovoked VTE [16].

Aspirin is associated with risk of GI toxicity, leading to ulceration and bleeding. For patients taking aspirin, who are at risk of GI events, concomitant use of proton pump inhibitor (PPI) is currently recommended. However, PPI is under-prescribed in these patients [17]. In the MAGIC study, patients receiving PPI had lower risk of GI ulcer or erosion [9]. Therefore, PPI should be more widely used concomitantly with aspirin to reduce GI toxicity for long-term prevention of CV events in patients with history of CV diseases.

As study limitations, systematic screening was not conducted in each hospital for patient recruitment. Our registry recruited patients receiving low-dose aspirin who were at high risk of CV events in the routine clinical practice of selected sites. Inclusion bias may be a potential limitation of this study. In addition, the MAGIC study was a prospective observational registry, but the events were judged by investigators at individual sites and not by a central Event Adjudication Committee, which was not set up in this study. Obviously, our study is not a randomized controlled trial. Although we have a monitoring committee overseeing our registry, the quality of our registry data is not in compliance with GCP.

## Conclusions

Despite various limitations, we believe that the MAGIC study prospectively suggests the risk of CV and major bleeding events at 1-year follow-up in patients with a history of CV disease who were given aspirin. The results of the MAGIC study may provide important data for future clinical trials and may be refined by a future high-quality database.
